# Simian Varicella Virus Infects Enteric Neurons and α4β7 Integrin-Expressing Gut-Tropic T-Cells in Nonhuman Primates

**DOI:** 10.3390/v10040156

**Published:** 2018-03-28

**Authors:** Werner J. D. Ouwendijk, Suzanne van Veen, Tamana Mehraban, Ravi Mahalingam, Georges M. G. M. Verjans

**Affiliations:** 1 Department of Viroscience, Erasmus MC, 3015 CE Rotterdam, The Netherlands; S.van_Veen@lumc.nl (S.v.V.); t.mehraban@erasmusmc.nl (T.M.); g.verjans@erasmusmc.nl (G.M.G.M.V.); 2 Department of Neurology, University of Colorado School of Medicine, Anschutz Medical Campus, Aurora, CO 80045, USA; Ravi.Mahalingam@ucdenver.edu; 3 Research Center for Emerging Infections and Zoonoses, University of Veterinary Medicine Hannover, 30559 Hannover, Germany

**Keywords:** varicella-zoster virus, simian varicella virus, enteric nervous system, nonhuman primate, T-cells

## Abstract

The pathogenesis of enteric zoster, a rare debilitating complication of reactivation of latent varicella-zoster virus (VZV) in the enteric nervous system (ENS), is largely unknown. Infection of monkeys with the closely related *Varicellovirus* simian varicella virus (SVV) mimics VZV disease in humans. In this study, we determined the applicability of the SVV nonhuman primate model to study *Varicellovirus* infection of the ENS. We confirmed VZV infection of the gut in latently infected adults and demonstrated that SVV DNA was similarly present in gut of monkeys latently infected with SVV using quantitative real-time PCR. In situ analyses showed that enteric neurons expressed SVV open reading frame (ORF) 63 RNA, but not viral nucleocapsid proteins, suggestive of latent ENS infection. During primary infection, SVV-infected T-cells were detected in gut-draining mesenteric lymph nodes and located in close vicinity to enteric nerves in the gut. Furthermore, flow cytometric analysis of blood from acutely SVV-infected monkeys demonstrated that virus-infected T-cells expressed the gut-homing receptor α4β7 integrin. Collectively, the data demonstrate that SVV infects ENS neurons during primary infection and supports the role of T-cells in virus dissemination to the gut. Because SVV reactivation can be experimentally induced, the SVV nonhuman primate model holds great potential to study the pathogenesis of enteric zoster.

## 1. Introduction

Most adults worldwide are infected with the human neurotropic *Varicellovirus* varicella-zoster virus (VZV) [1]. Primary VZV infection causes varicella and leads to establishment of a lifelong latent infection in neurons of sensory and autonomic ganglia, including those of the enteric nervous system (ENS) [1,2]. Reactivation of latent VZV from sensory ganglia causes herpes zoster (HZ), frequently accompanied by post-herpetic neuralgia [1]. VZV may also reactivate in the ENS, mainly in immunocompromised individuals, leading to gastrointestinal (GI) dysfunction associated with virus replication in the gut in the absence of skin rash [2,3]. The pathogenesis of enteric zoster, associated with intestinal pseudo-obstruction and gastric ulcers, is largely unknown.

VZV infection of the ENS has been studied in ante- and postmortem human intestine biopsies, and more thoroughly in the guinea pig model of VZV infection [3,4,5,6,7]. VZV inoculation of guinea pigs leads to infection and viral persistence in ENS neurons [4], and virus reactivation can be induced in vitro using VZV-infected isolated guinea pig ENS neurons [3,4]. Furthermore, the guinea pig model provided essential insights into the route by which VZV could spread to the ENS [5]. However, guinea pigs are not the natural host of VZV and reactivation of VZV from sensory or ENS neurons has not been described. Pathogenesis of the nonhuman primate *Varicellovirus*, simian varicella virus (SVV), closely mimics the immunological and clinical features of VZV infection in humans, including varicella, lifelong neuronal latency and (experimentally induced) HZ [8]. Herein, we determined the applicability of the SVV nonhuman primate model to study *Varicellovirus* infection of the ENS.

## 2. Materials and Methods

### 2.1. Tissue Specimens from Nonhuman Primates

Formalin-fixed and paraffin-embedded (FFPE) tissues were obtained from two Chinese rhesus macaques (RM) intratracheally infected with wild-type SVV (SVV.wt; RM 2207 and 9021) at 21 days post-infection (dpi), and African green monkeys (AGM) intratracheally infected with SVV.wt (AGM 269 and 279) or recombinant SVV expressing enhanced green fluorescent protein (SVV.EGFP; AGM 273 and 294) at 9 dpi (AGM 269 and 294) and 13 dpi (AGM 273 and 279). The experimental procedures on the animals analyzed were reported previously [9,10]. RM 2207 and 9021 developed robust viremia during acute infection [9]. At 21 dpi, viral DNA and restricted viral gene expression were detected in ganglia, but not in lung, liver or spleen, suggesting that SVV latency was established [9]. All AGM developed a pronounced viremia, pneumonitis, fever and skin rash during acute infection [10]. Acute and latent SVV infections were analyzed in AGM and RM, respectively, because surplus intestine material was available from previous studies [9,10]. Animals were housed and experiments were performed in compliance with European guidelines (EU Directive on Animal Testing 86/609/EEC) and Dutch legislation (Experiments on Animals Act, 1997). The protocol was approved by the independent animal experimentation ethical review committee DCC (27 June 2011, Erasmus MC permit number EMC2374).

### 2.2. DNA Extraction from Human Specimens

Archived snap-frozen human intestine specimens, obtained for diagnostic purposes, were kindly provided by the Erasmus MC Tissue Bank (Rotterdam, The Netherlands) (Table 1). Material was obtained in accordance with the institutional rules and regulations, as described [11]. From each biopsy, 25 consecutive cryosections (10 µm thick) were cut and used for DNA isolation (Sections 1–20) using the QIAmp DNA Mini Kit (Qiagen, Hilden, Germany) and immunohistochemical (IHC) staining (Sections 21–25) [9,10].

### 2.3. DNA Extraction from Rhesus Macaque Intestine

DNA was isolated from ten consecutive FFPE intestine sections (10 µm thick) of RMs using the QIAamp DNA FFPE Tissue Kit (Qiagen). For some experiments, DNA was isolated from pooled sections from colon, duodenum, ileum, jejunum and cecum of the same animal, while in other experiments all sections were obtained from the same tissue block (Table 2).

### 2.4. Quantitative Real-Time PCR

Quantitative real-time PCR (qPCR) was performed in duplicate on DNA using Taqman 2× PCR Universal Master Mix (Applied Biosystems, Foster City, CA, USA) with primers and probes specific for herpes simplex virus 1 (HSV-1) unique short gene 4 (*US4*), VZV open reading frame 62 (ORF62), SVV ORF21, and the human and nonhuman primate genes hydroxymethylbilane synthase (HMBS) and oncostatin-M (OSM) as described [10,12,13]. Commercially available HSV-1 and VZV DNA stocks (Advanced Biotechnologies, Eldersburg, MD, USA), and plasmids expressing SVV ORF21, were used to standardize qPCR reactions and used as positive control in each qPCR assay [10,12,13]. HMBS and OSM were used as endogenous controls for DNA integrity and quantification of number nucleated host cells in sections used for DNA extraction [10,12,13]. Lower limit of detection was 3 viral genome copies/reaction for HSV-1 DNA, 1 copy/reaction for VZV DNA, 1 copy/reaction for SVV DNA, 10 cells/reaction for HMBS and 6 cells/reaction for OSM (Supplementary Figure S1). The following primers and probes were used: HBMS-probe (6-FAM–TGG-AAG-CTA-ATG-GGA-AGC-CCA-GTA-CC–TAMRA), HMBS-Fw (GCC-TGC-AGT-TTG-AAA-TCA-GTG), HMBS-Rv (CGG-GAC-GGG-CTT-TAG-CTA), VZV ORF62-probe (6-FAM–TGC-AAC-CCG-GGC-GTC-CG–BHQ1), VZV ORF62-Fw (CCT-TGG-AAA-CCA-CAT-GAT-CGT), VZV ORF62-Rv (AGC-AGA-AGC-CTC-CTC-GAC-AA), HSV-1 US4-probe (6-FAM–CGT-CTG-GAC-CAA-CCG-CCA-CAC-AGG-T–BHQ1), HSV US4-Fw (TCC-TG/CG-TTC-CTA/C-ACG/T-GCC-TCC-C), HSV US4-Rv (GCA-GIC-AC/TA-CGT-AAC-GCA-CGC-T), SVV ORF21-probe (6-FAM–TCC-ATC-CTG-AAC-GAT-AGG-CAT-GTC-ATA-AAG-A–BHQ1), SVV ORF21-Fw (GAC-ACA-TCA-GCG-GTT-TGC-A), SVV ORF21-Rv(TGC-ACG-CTG-TGT-TAG-AAT-TCG), OSM-probe (6-FAM–TAC-TGC-ATG-GCC-CAG-CTG-CTG-GAC-AA–BHQ1), OSM-Fw (CCT-CGG-GCT-CAG-GAA-CAA-C) and OSM-Rv (GGC-CTT-CGT-GGG-CTC-AG).

### 2.5. In Situ Analyses

Deparaffinized and rehydrated intestinal tissue sections (4 µm thick) were subjected to heat-induced antigen retrieval in citrate buffer, blocked and incubated overnight at 4 °C with the following primary antibodies: mouse anti-human neural cell adhesion molecule (NCAM) (clone 123C3.D5; ThermoFisher Scientific, Waltham, MA, USA), mouse anti-CD3 (F7.2.38; Dako, Troy, MI, USA), rabbit anti-SVV nucleocapid proteins (SVV-NP) [10] or appropriate isotype control antibodies. Sections were incubated with biotinylated secondary goat anti-rabbit Ig or anti-mouse Ig antibodies, followed by streptavidin-conjugated horseradish peroxidase (all from Dako) and the immunohistochemistry (IHC) signal visualized using 3-amino-9-ethylcarbazole. Nuclei were counterstained with hematoxylin (Sigma, Kawasaki Shi, Japan) [10]. Immunofluorescent staining was visualized using Alexa Fluor 488- and Alexa Fluor 594-conjugated goat anti-rabbit and anti-mouse secondary antibodies, nuclei were stained with Hoechst 33342 and sections were mounted with Prolong Diamond Antifade medium (ThermoFisher Scientific) [10]. Confocal microscopy images were obtained using a Zeiss LSM 700 confocal laser scanning microscope. ZEN 2010 software (Zeiss, Oberkochen, Germany) was used to adjust brightness and contrast.

In situ hybridization (ISH) was performed using the RNAscope 2.0 kit red (Advanced Cell Diagnostics, ACD, Segrate, Italy) and oligonucleotide probes directed to SVV ORF63, human ubiquitin C (UBC; positive control), the bacterial dihydrodipicolinate reductase gene (DapB; negative control) and VZV ORF63 (negative control) were designed and obtained from ACD. Sections were counterstained with hematoxylin and mounted in Ecomount (Biocare Medical, Pacheco, CA, USA). The highly sensitive RNAScope methodology (Advanced Cell Diagnostics) enables in situ detection of single RNA molecule visualized as a punctuate signal dot in tissue sections with a standard microscope [14].

### 2.6. Flow Cytometry

Peripheral blood mononuclear cells (PBMC) from three SVV.EGFP-infected AGM obtained at 0, 5, 7 and 9 dpi were stained with fluochrome-conjugated antibodies directed to human CD28 (clone CD28.2), CD69 (FN50), CD95 (DX-2), CD3 (SP34-2), CD4 (L200), CD8 (SK1) (all from BD Biosciences), and macaque α4β7 integrin (NIH nonhuman primate reagent resource), as described [10]. Cells were measured on a FACS Canto II (BD Biosciences, Franklin Lakes, NJ, USA) and analyzed using FlowJo software (Tree Star Inc., Ashland, OR, USA).

### 2.7. Statistical Analysis

Statistical differences were analyzed in GraphPad Prism 5 (GraphPad Software, Inc., San Diego, CA, USA).

## 3. Results

### 3.1. Detection of VZV DNA in Human Intestine Biopsies

To determine VZV infection of the human GI tract [3,4,5], 18 human snap-frozen intestine biopsies were analyzed for the presence of VZV DNA, and as a control the related human alphaherpesvirus HSV-1 DNA, by qPCR (Table 1). Ten of 18 (56%) of individuals were female, median age at time of biopsy was 57.5 years (range: 32–75), 17 of 18 (94%) patients had cancer and none of the patients had VZV-related disease at the time of biopsy. All biopsies contained enteric ganglia, as determined by IHC for NCAM expression. Whereas HSV-1 was undetectable, VZV DNA was found in intestine biopsies of 5 of 18 (28%) individuals [median of 93 VZV genome copies per 100,000 cells (range: 2–423)] (Table 1). Thus, VZV but not HSV-1 DNA is present in human intestine biopsies, confirming previous studies on latent VZV infection of the ENS [3,4,5].

### 3.2. Detection of Viral DNA and RNA in Intestine of Latently SVV-Infected Rhesus Macaques

**Figure 1:**
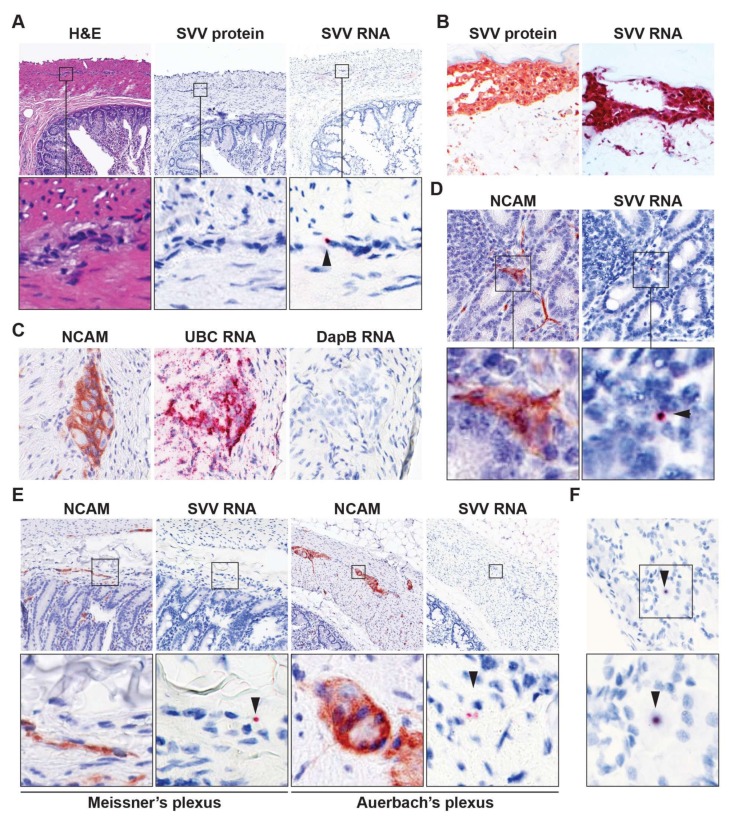
Detection of viral RNA in enteric neurons of latently simian varicella virus (SVV)-infected rhesus macaques. (**A**) Consecutive intestine sections from latently SVV-infected rhesus macaques (RM) were stained with hematoxylin and eosin (H&E), immunohistochemically (IHC) stained for SVV nucleocapsid protein or stained for SVV ORF63 RNA by in situ hybridization (ISH). Note punctate red ISH staining (arrow head), indicative of low level ORF63 expression. Magnification: 100×; inset: 1000×; (**B**) Validation of SVV-specific IHC and ISH staining in varicella skin biopsies from SVV-infected African green monkeys [AGMs 273 (IHC) and 269 (ISH)]. Magnification: 200×; (**C**) Consecutive intestine sections from latently SVV-infected RM were assayed for NCAM expression by IHC, and RNA expression of ubiquitin C (human cellular UBC gene; positive control) and DapB (bacterial gene, negative control) by ISH. Representative images are shown for RM 9021. Magnification: 400×; (**D**,**E**) Consecutive RM intestine sections were stained for NCAM by IHC and SVV ORF63 RNA by ISH. Three SVV DNA qPCR^P^°^S^ intestine paraffin blocks (each containing three distinct biopsies) from two RM (Table 2) were analyzed; (**F**) Representative images of a SVV ORF63 ISH^P^°^S^ neuron in latently SVV-infected lumbar dorsal root ganglia of RM 2207; (**D**,**F**), magnification: 400×; (**E**) magnification: 200×. Inset shows enlargement of area indicated by the black box. Arrowheads indicate SVV ISH^P^°^S^ neurons. Representative images of SVV ISH^P^°^S^ enteric ganglia (on average 4.3 ISH^P^°^S^ ganglia per section) are shown for RM 2207 (**A**,**E**); and SVV ISH^P^°^S^ nerve fibers (on average 14.3 ISH^P^°^S^ nerve fibers per section) are shown for RM 9021 (**D**).

To study the establishment of SVV latency in the ENS of SVV-infected nonhuman primates, we first determined the presence of SVV DNA in FFPE intestine tissue sections of two latently SVV.wt-infected RM (RM 2207 and 9021 [9]). Low amounts of SVV DNA were detected in the gut of both animals, without any apparent predominance of SVV infection for specific GI compartments (Table 2). Next, we determined the virus-infected cell types in SVV DNA^P^°^S^ intestine sections by SVV-specific IHC and ISH analyses. Like VZV [12,15], low SVV ORF63 transcript levels are frequently detected in sensory ganglia of latently SVV-infected animals [16]. Whereas no SVV-NP expression could be detected by IHC, SVV ORF63 RNA was detected in cells morphologically resembling neurons of enteric ganglia (Figure 1A). In contrast, both SVV-NP and viral RNA were readily detected in varicella skin sections of acutely infected animals (Figure 1B). Parallel ISH analysis using positive (human UBC gene) and negative (bacterial DapB gene) control probes clearly demonstrated the high sensitivity and specificity of the ISH platform used (Figure 1C). Furthermore, we did not detect specific ISH signal in gut of latently SVV-infected RM using probes directed to VZV ORF63 or in gut sections of uninfected cynomolgus macaques stained for SVV ORF63 (Supplementary Figure S2). To determine SVV RNA expression in neurons, consecutive sections were stained for NCAM protein and SVV ORF63 RNA by IHC and ISH, respectively. SVV ORF63 RNA expression appeared to co-localize with NCAM-positive cells, most likely nerve fibers, dispersed throughout the intestinal mucosa and submucosa (Figure 1D). Low SVV ORF63 RNA expression was also detected in areas containing neuronal cell bodies in both the submucosal (Meissner’s) and myenteric (Auerbach’s) plexuses (Figure 1E). Note that the SVV ORF63 RNA expression levels in the ENS (Figure 1D) resembled that of latently SVV-infected dorsal root ganglion (DRG) neurons in the same animal (Figure 1F). These data demonstrate that SVV infects, and potentially establishes latency, in ENS cells following primary infection of nonhuman primates.

### 3.3. SVV Infects T-Cells in Mesenteric Lymph Nodes

**Figure 2:**
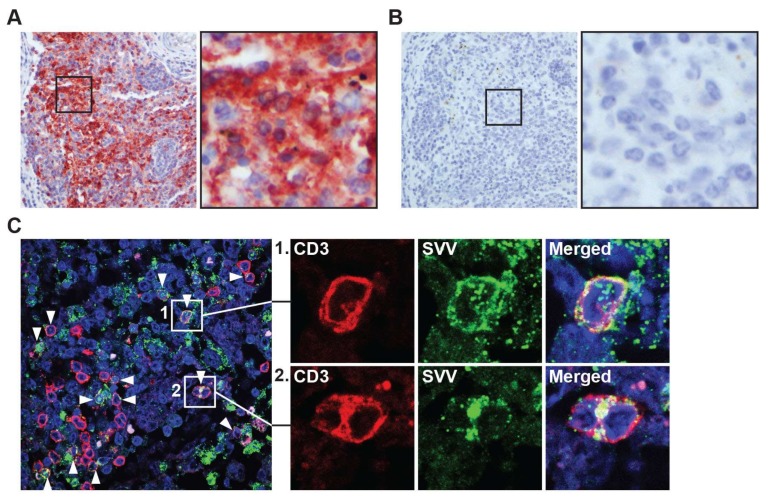
Detection of SVV in intestine-draining mesenteric lymph nodes during acute SVV infection of African green monkeys. (**A**,**B**) Consecutive sections of mesenteric lymph nodes, obtained from SVV-infected African green monkeys (AGM) at 9 dpi (acute infection), were stained for by IHC using rabbit anti-SVV nucleocapsid (SVV-NP) (**A**) and normal rabbit control serum (**B**); Magnification: 200×; inset: 1000×; (**C**) Sections of mesenteric lymph nodes obtained from SVV-infected AGM at 9 dpi were assayed by immunofluorescence for expression of CD3 (red) and SVV-NP (green), and nuclei were counterstained with Hoechst 33342 (blue). Arrowheads indicate examples of SVV-infected T-cells. Magnification: 400×. Inset shows enlargement of area indicated by the white boxes. Representative images shown for AGM 269.

Next, we investigated the potential role of T-cells in the dissemination of SVV to the GI tract. During primary infection, SVV and VZV spread to lymph nodes draining the respiratory tract, where virus is relayed to T-cells that subsequently disseminate the virus to skin and ganglia [10,17,18,19]. Mesenteric lymph nodes (mLN) drain the intestine and contain high numbers T-cells that can traffic to the gut [20]. To investigate whether SVV infects mLN during primary infection, we analyzed mLN from SVV-infected AGM at 9 and 13 dpi. Abundant SVV antigen was detected in mLN at 9 dpi (2 days after peak viremia [10]) of both SVV.wt- and SVV.EGFP-infected AGM (Figure 2A,B), and to a lesser extend in mLN of SVV.wt-infected AGM at 13 dpi (6 days after peak viremia [10]). Notably, SVV-infected T-cells were abundantly detected in mLN at 9 dpi using double-immunofluorescent staining for CD3^P^°^S^ T-cells and SVV-NP (Figure 2C).

### 3.4. SVV Infects Gut-Tropic T-Cells In Vivo

**Figure 3:**
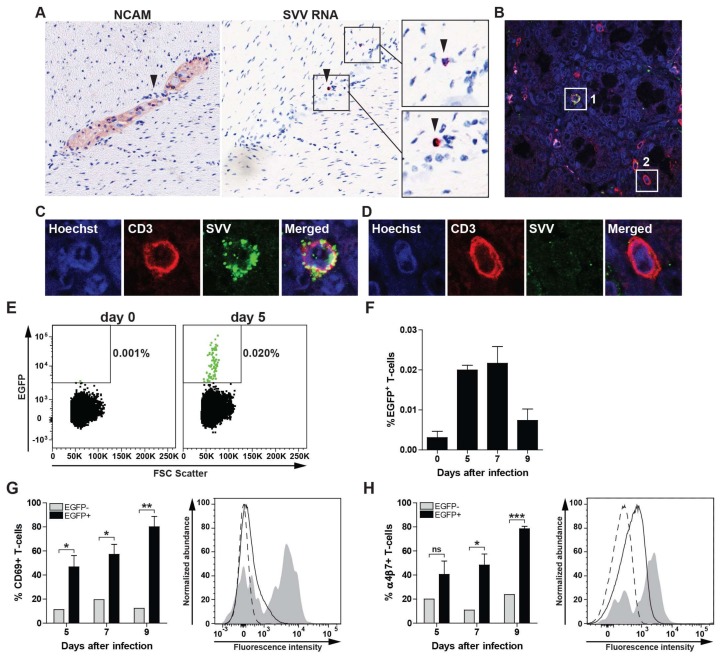
Expression of CD69 and α4β7 integrin on SVV-infected T-cells during acute infection of African green monkeys. (**A**) Consecutive intestine sections from SVV-infected African green monkeys (AGM) at 9 days post infection (dpi) were stained for NCAM by IHC and SVV ORF63 by ISH. SVV-infected cells localized in close vicinity to enteric neurons in the absence of viral RNA expression in neurons. Magnification: 200×; Inset: 1000×. Arrowheads indicate same cell in adjacent sections; (**B**–**D**) Confocal microscopy image showing co-localization of SVV antigen (green) and CD3 (red) in intestine sections from SVV-infected AGM at 9 dpi. Nuclei were counterstained using Hoechst 33342 (blue). Magnification: 400×; (**C**,**D**) Show enlargements of white boxes indicated with #1 (example of SVV-infected T-cell) and #2 (uninfected T-cell). Two intestine paraffin blocks (each containing four distinct biopsies) from two AGM were analyzed. Number of ORF63 ISH^P^°^S^ lymphocyte-like cells and SVV-infected T-cells ranged in numbers from scarce (AGM 294) to abundant (AGM 269). Representative images are shown for AGM 294 (**A**) and AGM 269 (**B**–**D**) [10]; (**E**,**F**) Peripheral blood mononuclear cells (PBMC) from a SVV.EGFP-infected AGM were analyzed for the presence of SVV-infected (EGFP^+^) CD3^+^ T-cells by flow cytometry; (**E**) Dot plots showing EGFP^+^ events (green) among 100,000 T-cells at 0 and 5 dpi for AGM 294; (**F**) Frequency of EGFP^+^ T-cells in three SVV.EGFP-infected AGM at 0, 5, 7 and 9 dpi; (**G**,**H**) CD69 (**G**) and α4β7 integrin (**H**) expression on SVV.EGFP-infected (EGFP+) and uninfected (EGFP−) CD3+ T-cells from three SVV.EGFP-infected AGM at 0, 5, 7 and 9 dpi. Histograms show the expression or CD69 (**G**) and α4β7 integrin (**H**) on uninfected (EGFP−, solid black line) or SVV-infected (EGFP+) PBMC (grey filled) of AGM 273 at 5 dpi [10]. Dashed black line indicates fluorescence minus one (FMO) control; (**F**–**H**) Data represent average ± SEM from 3 AGM. ns: not significant; * *p* <0.05, *** *p* < 0.001 by unpaired Student’s *t*-test.

To determine the presence of SVV-infected lymphocytes in the gut during primary infection, consecutive intestine sections from SVV-infected AGMs obtained at 9 dpi were stained by IHC for NCAM and by ISH for SVV ORF63 RNA. Detection of SVV RNA in small lymphocyte-like cells in close proximity to enteric ganglion neurons suggests viremic spread of SVV to the ENS (Figure 3A). Consistent with detection of SVV-infected T-cells in mLN, we detected gut-infiltrating SVV-infected T-cells in the gut of acutely infected AGM by double-immunofluorescent staining (Figure 3B,C) as well as uninfected T-cells (Figure 3D).

Previous studies showed that VZV infection reconfigures human T-cells to become activated (CD69^+^), skin-tropic (CLA^+^CCR4^+^) T-cells [18,21,22]. Homing of T-cells to the gut is mediated by the expression of the α4β7 integrin on T-cells and its ligand mucosal vascular addressing cell-adhesion molecule 1 (MADCAM1) on endothelial cells in the intestine [23,24]. Therefore, we analyzed expression of CD69 and α4β7 integrin on SVV-infected T-cells in SVV.EGFP-infected AGMs at 5, 7 and 9 dpi (peak viremia ± 2 days) [10]. Low frequencies of SVV.EGFP-infected (EGFP^+^) T-cells were readily detected in blood at 5, 7 and 9 dpi (Figure 3E,F). Analogous to the increased CD69 expression on in vitro VZV-infected human T-cells [21,22], SVV-infected T-cells were highly enriched for CD69 expression in blood of acutely SVV-infected AGM (Figure 3G). Moreover, these T-cells also expressed α4β7 integrin (Figure 3H), suggesting that T-cells contribute to viremic spread of SVV to the ENS during primary infection.

## 4. Discussion

In addition to varicella and HZ [25], VZV is implicated in a broad spectrum of neurological, vascular and gastrointestinal diseases [1]. The pathogenesis underlying complications of VZV reactivation is incompletely understood, largely due to the lack of small animal models that closely mimic human VZV infection [1,2,8]. In this study, we report that SVV infects enteric neurons and gut-tropic T-cells in nonhuman primates during primary infection, supporting the applicability of the SVV nonhuman primate model to study *Varicellovirus* infection of the ENS.

We confirmed that VZV DNA, but not HSV-1 DNA, is present in gut biopsies of human adults without herpesvirus-related diseases at the time of biopsy. Unfortunately, we did not have access to blood samples to analyze potential subclinical VZV reactivation. We report lower frequencies of VZV DNA^P^°^S^ intestine biopsies (28%) compared to earlier reports (>85%) [3,4,5], most likely due to the limited quantity of tissue available in our study. While our analyses were restricted to only twenty 10 µm-thick sections, obtained from gut biopsies with an average diameter of 5 mm, previous studies used complete surgical gut specimens for PCR analysis to detect low amounts of VZV DNA and/or RNA [3,4,5,26]. Furthermore, our data implicate that HSV-1 does not persist in enteric neurons of the human gut.

SVV reactivation is associated with lesions in the GI tract in severely immunocompromised nonhuman primates, suggesting that SVV can also establish latency and reactivate from the ENS [27,28]. Herein, we demonstrated that SVV infect ENS neurons upon primary infection. The low quantities of viral DNA and ORF63 RNA detected by qPCR and ISH, and absence of detectable levels SVV-NP expression by IHC in intestine of SVV-infected RM at 21 dpi, suggest that SVV can establish latency in the ENS. However, these observations need to be confirmed in future studies using larger cohorts and at later stages after primary SVV infection to demonstrate true viral latency in enteric neurons.

The neuronal cell types harboring latent virus and mechanisms underlying VZV latency in the ENS are incompletely understood. The ENS is composed of a complex network of diverse neuron subtypes that involve both sympathetic and parasympathetic motor and sensory neurons [2]. The low quantities of VZV DNA detected (Table 1) and reported by others [3,4,5,26], and the low number of enteric neurons expressing SVV ORF63 RNA at 21 dpi, suggest that infection of ENS neurons occurs at low frequency or is restricted to specific enteric neuron subtypes, as described for VZV infection in human fetal DRG [29]. In contrast to VZV latency in human sensory ganglia [12,30,31], latently VZV-infected enteric neurons in the guinea pig model express a restricted set of viral transcripts and proteins in [5,32]. The SVV nonhuman primate model provides the ideal platform to define and compare the characteristics of viral latency in enteric ganglia versus the classical sites of *Varicellovirus* latency, including DRG and trigeminal ganglia in the same animal.

The route by which SVV and VZV infect enteric ganglion neurons is unclear. SVV and VZV may infect enteric neurons by the transaxonal route, via projections of DRG neurons that innervate both the epidermis and viscera [5], and/or by the hematogenous route involving T-cells as previously shown for skin and ganglia [10,17,18,32]. The detection of SVV-infected α4β7^+^ T-cells in blood and their localization in close vicinity to enteric neurons during primary SVV infection supports the role of T-cells in SVV dissemination to the ENS in nonhuman primates. However, it is unclear if the virus preferentially infects α4β7^+^ T-cells, e.g., located in mLN [20], or induces its expression upon infection. Recent studies showed that VZV infection remodels human T-cell to become activated, skin-tropic memory T-cells [21,22]. Whereas β7 integrin expression was not investigated, VZV-induced expression of α4 integrin (CD49d) on infected human T-cells suggest a similar mode of action [22]. Furthermore, similar to the in vitro observed upregulation of CD69 expression in VZV-infected T-cells [21], we observed increased CD69 expression by SVV-infected T-cells in vivo. These findings suggest that both VZV and SVV modulate the phenotype and thereby tissue-tropism of T-cells upon infection.

In conclusion, the data demonstrate that the SVV nonhuman primate model is of additive value in future studies aimed to elucidate the pathogenesis of *Varicellovirus* infection of the ENS. Compared to the VZV guinea pig model [5], the SVV model has the unique advantage to study the virus/host interactions of a species-restricted *Varicellovirus*, adapted to nonhuman primates during long-term co-specification, and the ability to experimentally induce reactivation resembling HZ in humans [8]. The worldwide use of the live-attenuated vOka varicella vaccine, which establishes latency in ENS neurons of vaccinated children and may reactivate later in life causing GI dysfunction [3,26], warrants the development of novel intervention strategies that prevent the complete spectrum of VZV-related disease including enteric zoster.

## Figures and Tables

**Table 1:** Detection of varicella-zoster virus DNA in human intestine biopsies.

Patient ID	Gender ^1^	Age ^2^	Disease at Time of Intestinal Biopsy	DNA ^3^	
				HSV-1	VZV
1	F	72	Small intestine B-cell lymphoma	–	2
2	F	55	Small intestine neuroendocrine tumor	–	107
3	F	53	Colonic adenocarcinoma	–	–
4	F	57	Small intestine B-cell lymphoma	–	–
5	F	51	Pancreatic carcinoma	–	–
6	M	44	Small intestine gastrointestinal stromal tumor	–	–
7	F	40	Colonic adenocarcinoma	–	–
8	F	65	Bladder adenocarcinoma	–	–
9	M	48	Metastatic melanoma	–	–
10	M	61	Small intestine neuroendocrine tumor	–	–
11	F	64	Ovarian papillary serous carcinoma	–	423
12	F	60	Colonic adenocarcinoma	–	–
13	M	58	Small intestine neuroendocrine tumor	–	–
14	M	55	Small intestine neuroendocrine tumor	–	–
15	M	73	Metastatic carcinoma (unknown origin)	–	–
16	F	75	Adnexal carcinosarcoma	–	93
17	M	32	Peutz-Jeghers syndrome	–	30
18	M	68	Carcinoid small intestine	–	–

^1^ F, Female; M, male; ^2^ Age in years; ^3^ Frozen human intestine biopsies were analyzed for presence of herpes simplex virus type 1 (HSV-1) and varicella-zoster virus (VZV) DNA by quantitative real-time PCR (qPCR). Viral genome copies per 100,000 human cells are indicated. –, undetectable by qPCR. Human genomic DNA was readily detected in all samples, as determined by qPCR for the human single copy gene hydroxymethylbilane synthase [HMBS, average (±SEM) C_t_-value = 25.4 (±0.23)].

**Table 2:** Detection of simian varicella virus DNA in intestine of latently infected rhesus macaques.

Animal ID	Tissue ^1^	SVV DNA ^2^
9021	Colon + small intestine	14
	Colon	11
	Duodenum	–
	Ileum	–
	Jejunum	–
	Caecum	–
2207	Colon + small intestine	290
	Colon	–
	Duodenum	12
	Ileum	14
	Jejunum	–
	Caecum	110

^1^ DNA was extracted from ten serial sections (10 µm thick) of formalin-fixed and paraffin-embedded intestine tissue blocks, either pooled sections from colon and small intestine, or individual intestine segments; ^2^ SVV DNA load was determined by SVV ORF21-specific qPCR analysis. SVV genome copies per 100,000 cells are indicated. –, undetectable by qPCR; Host gene oncostatin-M DNA was detected in all samples analyzed [average (±SEM) C_t_-value = 30.7 (±0.63)].

## Supplementary Materials

Supplementary materials can be found at http://www.mdpi.com/1999-4915/10/4/156/s1.

## References

[1] Gershon A.A., Breuer J., Cohen J.I., Cohrs R.J., Gershon M.D., Gilden D., Grose C., Hambleton S., Kennedy P.G.E., Oxman M.N. (2015). Varicella zoster virus infection. Nat. Rev. Dis. Prim..

[2] Rao M., Gershon M.D. (2016). The bowel and beyond: The enteric nervous system in neurological disorders. Nat. Rev. Gastroenterol. Hepatol..

[3] Gershon A.A., Chen J., Gershon M.D. (2015). Use of Saliva to Identify Varicella Zoster Virus Infection of the Gut. Clin. Infect. Dis..

[4] Gershon A.A., Chen J., Gershon M.D. (2008). A Model of Lytic, Latent, and Reactivating Varicella-Zoster Virus Infections in Isolated Enteric Neurons. J. Infect. Dis..

[5] Chen J.J., Gershon A.A., Li Z., Cowles R.A., Gershon M.D. (2011). Varicella zoster virus (VZV) infects and establishes latency in enteric neurons. J. Neurovirol..

[6] Carrascosa M.F., Salcines-Caviedes J.R., Román J.G., Cano-Hoz M., Fernández-Ayala M., Casuso-Sáenz E., Abascal-Carrera I., Campo-Ruiz A., Martín M.C., Díaz-Pérez A. (2014). Varicella-zoster virus (VZV) infection as a possible cause of Ogilvie’s syndrome in an immunocompromised host. J. Clin. Microbiol..

[7] Pui J.C., Furth E.E., Minda J., Montone K.T. (2001). Demonstration of varicella-zoster virus infection in the muscularis propria and myenteric plexi of the colon in an HIV-positive patient with herpes zoster and small bowel pseudo-obstruction (Ogilvie’s syndrome). Am. J. Gastroenterol..

[8] Ouwendijk W.J.D., Verjans G.M.G.M. (2015). Pathogenesis of varicelloviruses in primates. J. Pathol..

[9] Ouwendijk W.J.D., Mahalingam R., Traina-Dorge V., van Amerongen G., Wellish M., Osterhaus A.D.M.E., Gilden D., Verjans G.M.G.M. (2012). Simian varicella virus infection of Chinese rhesus macaques produces ganglionic infection in the absence of rash. J. Neurovirol..

[10] Ouwendijk W.J.D., Mahalingam R., de Swart R.L., Haagmans B.L., van Amerongen G., Getu S., Gilden D., Osterhaus A.D.M.E., Verjans G.M.G.M. (2013). T-Cell tropism of simian varicella virus during primary infection. PLoS Pathog..

[11] FEDERA Human Tissue and Medical Research: Code of Conduct for Responsible Use (2011). https://www.federa.org/sites/default/files/images/print_version_of_conduct_english.pdf.

[12] Ouwendijk W.J.D., Choe A., Nagel M.A., Gilden D., Osterhaus A.D.M.E., Cohrs R.J., Verjans G.M.G.M. (2012). Restricted varicella-zoster virus transcription in human trigeminal ganglia obtained soon after death. J. Virol..

[13] Van Velzen M., Ouwendijk W.J.D., Selke S., Pas S.D., van Loenen F.B., Osterhaus A.D.M.E., Wald A., Verjans G.M.G.M. (2013). Longitudinal study on oral shedding of herpes simplex virus 1 and varicella-zoster virus in individuals infected with HIV. J. Med. Virol..

[14] Wang F., Flanagan J., Su N., Wang L.C., Bui S., Nielson A., Wu X., Vo H.T., Ma X.J., Luo Y. (2012). RNAscope: A novel In Situ RNA analysis platform for formalin-fixed, paraffin-embedded tissues. J. Mol. Diagn..

[15] Cohrs R.J., Gilden D.H. (2007). Prevalence and abundance of latently transcribed varicella-zoster virus genes in human ganglia. J. Virol..

[16] Messaoudi I., Barron A., Wellish M., Engelmann F., Legasse A., Planer S., Gilden D., Nikolich-Zugich J., Mahalingam R. (2009). Simian varicella virus infection of rhesus macaques recapitulates essential features of varicella zoster virus infection in humans. PLoS Pathog..

[17] Zerboni L., Ku C.-C., Jones C.D., Zehnder J.L., Arvin A.M. (2005). Varicella-zoster virus infection of human dorsal root ganglia in vivo. Proc. Natl. Acad. Sci. USA.

[18] Ku C.-C., Zerboni L., Ito H., Graham B.S., Wallace M., Arvin A.M. (2004). Varicella-zoster virus transfer to skin by T Cells and modulation of viral replication by epidermal cell interferon-alpha. J. Exp. Med..

[19] Abendroth A., Morrow G., Cunningham A.L. (2001). Varicella-Zoster Virus Infection of Human Dendritic Cells and Transmission to T Cells: Implications for Virus Dissemination in the Host. J. Virol..

[20] Wang X., Xu H., Gill A.F., Pahar B., Kempf D., Rasmussen T., Lackner A.A., Veazey R.S. (2009). Monitoring α4β7 integrin expression on circulating CD4+ T cells as a surrogate marker for tracking intestinal CD4+ T-cell loss in SIV infection. Mucosal Immunol..

[21] Ku C.-C., Padilla J.A., Grose C., Butcher E.C., Arvin A.M. (2002). Tropism of varicella-zoster virus for human tonsillar CD4(+) T lymphocytes that express activation, memory, and skin homing markers. J. Virol..

[22] Sen N., Mukherjee G., Sen A., Bendall S.C., Sung P., Nolan G.P., Arvin A.M. (2014). Single-Cell Mass Cytometry Analysis of Human Tonsil T Cell Remodeling by Varicella Zoster Virus. Cell Rep..

[23] Berlin C., Berg E.L., Briskin M.J., Andrew D.P., Kilshaw P.J., Holzmann B., Weissman I.L., Hamann A., Butcher E.C. (1993). Alpha 4 beta 7 integrin mediates lymphocyte binding to the mucosal vascular addressin MAdCAM-1. Cell.

[24] Briskin M., Winsor-Hines D., Shyjan A., Cochran N., Bloom S., Wilson J., McEvoy L.M., Butcher E.C., Kassam N., Mackay C.R. (1997). Human mucosal addressin cell adhesion molecule-1 is preferentially expressed in intestinal tract and associated lymphoid tissue. Am. J. Pathol..

[25] Weller T.H., Witton H.M., Bell E.J. (1958). The etiologic agents of varicella and herpes zoster: Isolation, propagation, and cultural characteristics in vitro. J. Exp. Med..

[26] Gershon A.A., Chen J., Davis L., Krinsky C., Cowles R., Reichard R., Gershon M. (2012). Latency of varicella zoster virus in dorsal root, cranial, and enteric Ganglia in vaccinated children. Trans. Am. Clin. Climatol. Assoc..

[27] Gulani J., Koch A., Chappell M.G., Christensen C.L., Facemire P., Singh V.K., Ossetrova N.I., Srinivasan V., Holt R.K. (2016). Cercopithecine Herpesvirus 9 (Simian Varicella Virus) Infection after Total-Body Irradiation in a Rhesus Macaque (*Macaca mulatta*). Comp. Med..

[28] Kolappaswamy K., Mahalingam R., Traina-Dorge V., Shipley S.T., Gilden D.H., Kleinschmidt-Demasters B.K., McLeod C.G., Hungerford L.L., DeTolla L.J. (2006). Disseminated Simian Varicella Virus Infection in an Irradiated Rhesus Macaque (*Macaca mulatta*). J. Virol..

[29] Zerboni L., Arvin A. (2015). Neuronal Subtype and Satellite Cell Tropism Are Determinants of Varicella-Zoster Virus Virulence in Human Dorsal Root Ganglia Xenografts In Vivo. PLoS Pathog..

[30] Zerboni L., Sobel R.A., Lai M., Triglia R., Steain M., Abendroth A., Arvin A. (2012). Apparent expression of varicella-zoster virus proteins in latency resulting from reactivity of murine and rabbit antibodies with human blood group a determinants in sensory neurons. J. Virol..

[31] Ouwendijk W.J.D., Flowerdew S.E., Wick D., Horn A.K.E., Sinicina I., Strupp M., Osterhaus A.D.M.E., Verjans G.M.G.M., Hüfner K. (2012). Immunohistochemical detection of intra-neuronal VZV proteins in snap-frozen human ganglia is confounded by antibodies directed against blood group A1-associated antigens. J. Neurovirol..

[32] Gan L., Wang M., Chen J.J., Gershon M.D., Gershon A.A. (2014). Infected peripheral blood mononuclear cells transmit latent varicella zoster virus infection to the guinea pig enteric nervous system. J. Neurovirol..

