# Supplementary material for: Simian Varicella Virus Infects Enteric Neurons and α4β7 Integrin-Expressing Gut-Tropic T-Cells in Nonhuman Primates
Source: Viruses. 2018 Mar 28;10(4):156. doi: 10.3390/v10040156 (PMC5923450; doi:10.3390/v10040156)
Supplement: Supplementary file 1 [file viruses-10-00156-s001.pdf]

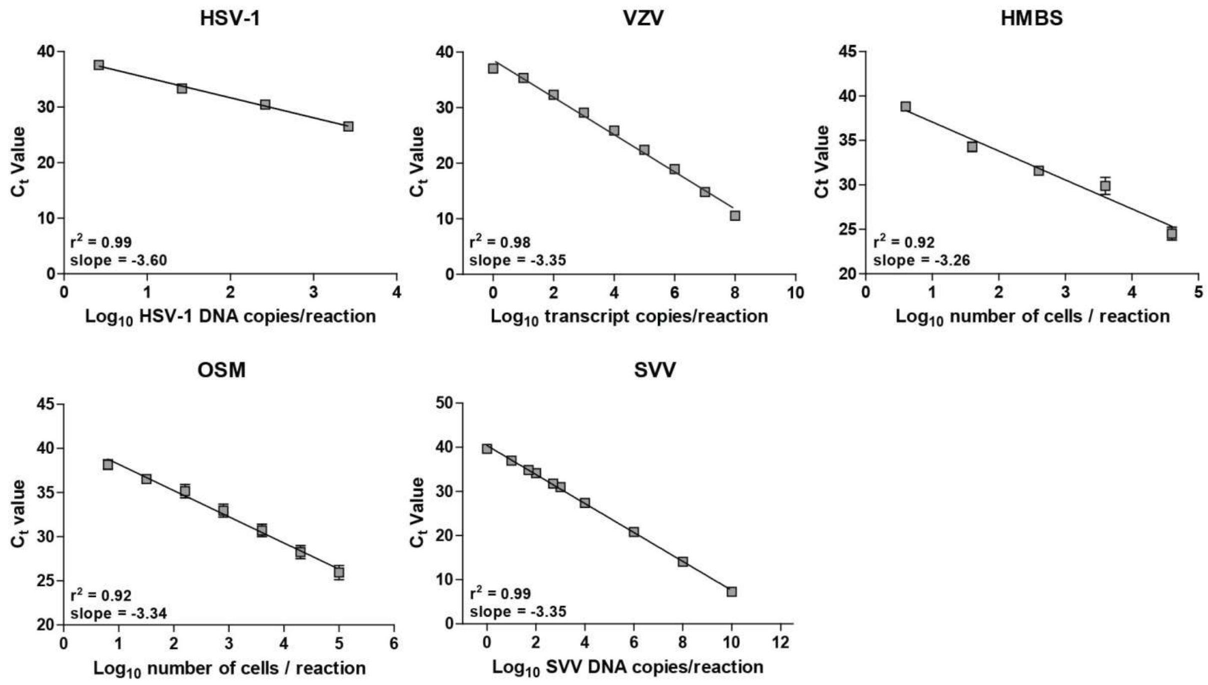

Supplementary Figure 1. Quantitative real-time PCR (qPCR) standard curves. The efficiency of HSV-1, VZV and SVV DNA quantitation was determined using serial dilutions of viral DNA diluted mixed with 100 ng of herring sperm DNA and primers and probes directed to HSV-1 US4, VZV ORF62 and SVV ORF21. Likewise, the efficiency of HMBS and OSM qPCRs to quantify human and nonhuman primate cells, respectively, was determined using DNA extracted from serial dilutions of cells. Each sample was measured in triplicate (viral DNA) or quadruplicate (host DNA) and error bars indicated standard error of the mean. Lines indicate linear regression curves, with slope and r^2-values indicated in each graph.

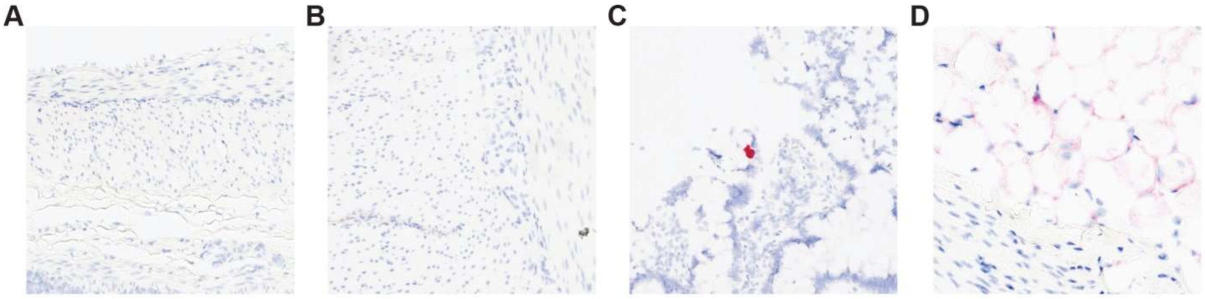

Supplementary Figure 2. Specificity of the *in situ* hybridization analyses. (**A**) Intestine section from latently SVV-infected rhesus macaques (RM)9021 stained for VZV ORF63 (negative control) by *in situ* hybridization (ISH). (**B**) Intestine section from an uninfected cynomolgus macaque stained for SVV ORF63 by ISH. (**C - D**) Examples of non-specific ISH staining in latently SVV-infected RM 9021 (**C**; substrate aggregate) and RM 2207 (**D**; non-specific diffuse staining of adipose tissue surrounding the intestine) stained for SVV ORF63 by ISH. (**A -C**), magnification: 200X. (**D**), magnification 600X.
